# Narrowband Blue and Red LED Supplements Impact Key Flavor Volatiles in Hydroponically Grown Basil Across Growing Seasons

**DOI:** 10.3389/fpls.2021.623314

**Published:** 2021-02-26

**Authors:** Hunter A. Hammock, Dean A. Kopsell, Carl E. Sams

**Affiliations:** Department of Plant Sciences, The University of Tennessee, Knoxville, Knoxville, TN, United States

**Keywords:** controlled environment, narrowband LEDs, flavor volatiles, *Ocimum basilicum*, secondary metabolism, supplemental lighting

## Abstract

The use of light-emitting diodes (LEDs) in commercial greenhouse production is rapidly increasing because of technological advancements, increased spectral control, and improved energy efficiency. Research is needed to determine the value and efficacy of LEDs in comparison to traditional lighting systems. The objective of this study was to establish the impact of narrowband blue (B) and red (R) LED lighting ratios on flavor volatiles in hydroponic basil (*Ocimum basilicum* var. “Genovese”) in comparison to a non-supplemented natural light (NL) control and traditional high-pressure sodium (HPS) lighting. “Genovese” basil was chosen because of its high market value and demand among professional chefs. Emphasis was placed on investigating concentrations of important flavor volatiles in response to specific ratios of narrowband B/R LED supplemental lighting (SL) and growing season. A total of eight treatments were used: one non-supplemented NL control, one HPS treatment, and six LED treatments (peaked at 447 nm/627 nm, ±20 nm) with progressive B/R ratios (10B/90R, 20B/80R, 30B/70R, 40B/60R, 50B/50R, and 60B/40R). Each SL treatment provided 8.64 mol ⋅ m^−2^ ⋅ d^–1^ (100 μmol ⋅ m^–2^ ⋅ s^–1^, 24 h ⋅ d^–1^). The daily light integral (DLI) of the NL control averaged 9.5 mol ⋅ m^−2^ ⋅ d^–1^ during the growth period (ranging from 4 to 18 mol ⋅ m^−2^ ⋅ d^–1^). Relative humidity averaged 50%, with day/night temperatures averaging 27.4°C/21.8°C, respectively. Basil plants were harvested 45 days after seeding, and volatile organic compound profiles were obtained by gas chromatography–mass spectrometry. Total terpenoid concentrations were dramatically increased during winter months under LED treatments, but still showed significant impacts during seasons with sufficient DLI and spectral quality. Many key flavor volatile concentrations varied significantly among lighting treatments and growing season. However, the concentrations of some compounds, such as methyl eugenol, were three to four times higher in the control and decreased significantly for basil grown under SL treatments. Maximum concentrations for each compound varied among lighting treatments, but most monoterpenes and diterpenes evaluated were highest under 20B/80R to 50B/50R. This study shows that supplemental narrowband light treatments from LED sources may be used to manipulate secondary metabolic resource allocation. The application of narrowband LED SL has great potential for improving overall flavor quality of basil and other high-value specialty herbs.

## Introduction

Plants have the ability to sense and respond to specific narrowband wavelengths within the ambient spectrum, ranging from ultraviolet-C (UV-C) (200–280 nm) to far-red (720–780 nm) regions (Galvao and Fankhauser, 2015; Carvalho et al., 2016). They use specialized pigment-proteins called photoreceptors, which react to changes in light intensity and spectra. This promotes physiological and biochemical changes that allow the plant to better adapt to the surrounding environment, further enhancing chances of survival/reproduction (Galvao and Fankhauser, 2015). Responses from abiotic stressors (i.e., changes in light intensity and spectra) prompt a diverse range of photomorphogenic responses across many plant species (Massa et al., 2008; Carvalho et al., 2016; Montgomery, 2016). Stimulation of phytochromes, cryptochromes, and phototropins has been shown to up- and down-regulate metabolic pathways that directly influence plant growth, development, and secondary metabolism (Briggs and Huala, 1999; Christie, 2007; Galvao and Fankhauser, 2015).

The solar radiation spectrum that has the most direct impact on plant growth and development includes three parts: UV light, visible (VS) light, and infrared (IR) light (McCree, 1973). Photosynthetically active radiation wavelengths (300–700 nm) are mostly absorbed by leaf tissues, and the majority of light absorption by chlorophyll pigments and quantum yield of photosynthesis occur primarily in the blue (B) and red (R) regions of the VS light spectrum (Morrow, 2008; Pimputkar et al., 2009; Kopsell et al., 2014). UV-C radiation includes 200–280 nm and is harmful to plants; however, UV-C is mostly blocked by the atmosphere, and most radiation of this type does not reach the earth’s surface. Lighting sources that produce high-level UV-C radiation may be harmful to plants/animals and should generally be avoided in horticulture applications. UV-B radiation includes 280–315 nm and is only harmful to plants under intense applications. UV-B exposure has the potential to increase secondary metabolite production, in addition to pigment bleaching and degradation of flavor volatiles/carotenoids/other secondary metabolites at intense levels. UV-A radiation includes 315–380 nm and has controversial impacts on plant morphology and secondary metabolite production. The range of 380–400 nm contains the transition from UV-A to the VS light spectrum. Chlorophyll and carotenoid pigments begin to absorb light within this range.

Wavelengths ranging from 400 to 520 nm contain violet, blue, and green light. Chlorophyll pigments obtain peak energy absorption at these wavelengths and strongly influence vegetative growth and development. Cryptochromes and phototropins are both B light receptors (Briggs and Christie, 2002). Cryptochromes act as signaling mechanisms that regulate circadian rhythms and prompt many physiological and morphological changes; phototropins control chloroplast movements to maximize absorption of specific wavelengths (Christie, 2007; Chaves et al., 2011; Kopsell et al., 2014). Changes in specific B wavelengths that target phototropins or cryptochromes have the ability to impact primary and secondary metabolism, volatile production, carotenoid and chlorophyll pigment bioaccumulation, circadian rhythms, stomatal opening/closing, intermodal length, leaf area and thickness, and intracellular structure configurations/positioning (Lichtenthaler, 1987; Frank and Cogdell, 1996; Briggs and Huala, 1999; Briggs and Christie, 2002; Christie, 2007; Abney et al., 2013; Kopsell and Sams, 2013; Metallo et al., 2018; Bantis et al., 2020; Samuoliene et al., 2020). Intensity changes to specific B wavelengths that target phytochromes have also been shown to impact germination rates, vegetative and reproductive growth/development, leaf size/thickness, phenolic and antioxidant pathways, etc. (Li and Kubota, 2009; Olle and Viršile, 2013; Landi et al., 2020). Exposure to UV light and specific B wavelengths have been shown to result in higher concentrations of favorable flavor volatiles in many high-value crops such as mint (*Mentha piperita*) (Lucchesi et al., 2004; Hikosaka et al., 2010; Treadwell et al., 2011), thyme (*Thymus vulgaris*) (Lee et al., 2005), strawberries *(Fragaria* × *ananassa)* (Colquhoun et al., 2013), chives (*Allium fistulosum*) (Abney et al., 2013), lettuce (*Lactuca sativa* L.) (Baumbauer et al., 2019; Chen et al., 2019; Kong et al., 2019; Yan et al., 2019; Zhang et al., 2019; Samuoliene et al., 2020), and basil (*Ocimum basilicum*) (Loughrin and Kasperbauer, 2003; Kopsell et al., 2005; Lee et al., 2005; Deschamps and Simon, 2006; Chalchat and Ozcan, 2008; Hussain et al., 2008; Klimánková et al., 2008; Bantis et al., 2016; Carvalho et al., 2016).

The range of 520–610 nm contains green, yellow, and orange wavelengths, which has limited influence on vegetative growth and development. Some studies have found that special wavebands within this range have specific impacts on plant development and secondary metabolite production, but additional research should be conducted to determine practical uses for specific wavebands within this range.

The range of 610–720 nm contains R wavelengths, and high levels of absorption occur at this range. These wavelengths strongly influence vegetative growth, photosynthesis, and reproductive growth. Phytochromes are mostly R light photoreceptors that control physiological responses via R and far-red wavelengths (Chaves et al., 2011). R light closely aligns with the maximum absorption for chlorophyll and matches the absorption peaks of various phytochemicals and secondary pigments (Goins et al., 1997; Pimputkar et al., 2009). Literature suggests that the interaction between cryptochromes/phytochromes/other accessory pigments and various wavelengths is not fully understood and should be researched further (Tennessen et al., 1994; Goins et al., 1997; Briggs and Huala, 1999; Morrow, 2008; Pimputkar et al., 2009; Chaves et al., 2011; Fraikin et al., 2013). Other unidentified photoreceptors may be influenced by the addition of these wavelengths.

The range of 720–1,000 nm contain far-red and IR wavelengths that directly impact germination and flowering. Wavelengths greater than 1,000 nm contain IR radiation, which is primarily converted into heat energy. These wavebands are not particularly useful to plants for primary or secondary metabolism and are not used for photosynthesis (McCree, 1973; Barta et al., 1992; Goins et al., 1997; Briggs and Huala, 1999; Lucchesi et al., 2004; Morrow, 2008; Abney et al., 2013; Tuan et al., 2013; Kopsell et al., 2014).

Secondary metabolites contain a wide range of chemical compound classes that serve many functions in plants, most of which involve adaptation to environmental stress (Bourgaud et al., 2001). In plant tissues, the synthesis of secondary metabolites is significantly impacted by environmental conditions in addition to many physiological, biochemical, and genetic factors (Bourgaud et al., 2001; Lefsrud et al., 2008). Light intensity and spectral quality are two of the most influential factors on secondary metabolism (Kopsell et al., 2005; Kopsell et al., 2014), and changes in light intensity and spectral quality directly impact plant physiology and biochemistry (Colquhoun et al., 2013; Bian et al., 2015; Landi et al., 2020).

Volatile organic compounds (VOCs) are defined as carbon-containing molecules that have high vapor pressure at ordinary room temperatures. Low boiling points, chemical structure, and other physical properties determine the high vapor pressures of volatile compounds (Taylor, 1996). These compounds include both naturally occurring and manmade compounds. A large number of scents, flavors, odors, and aromas are VOCs. The majority of VOCs found in nature are produced by plants, with isoprene being the base unit (i.e., isoprenoids). These compounds play an important role in plant signaling, defense, pollinator attraction, etc. (Kesselmeier and Staudt, 1999). Stabilization, volatilization, and plant-controlled emission of these compounds are directly impacted by genetics, as well as many abiotic and biotic factors such as temperature, sunlight, turgor pressure, stomatal opening, humidity, pest and pathogen pressures, etc. (Kesselmeier and Staudt, 1999; Klimánková et al., 2008). Emission occurs almost exclusively at the leaf’s surface through open stomata, but certain defense compounds may be released if physical damage occurs (Yousif et al., 1999). Aromatic and flavor compounds usually have low molecular weights (<300 Da), which directly contributes to their volatility along with chemical structure and high vapor pressures. Flavors affect both taste and smell, whereas fragrances (aroma) affect only smell (Fahlbusch et al., 2003). The terms *flavor* and *aroma* tend to denote naturally occurring compounds, whereas fragrance typically refers to synthetic compounds (Fahlbusch et al., 2003). Some studies have been performed on basil flavor volatiles, and substantial volatile profiles have been established using a variety of analytical techniques (Charles and Simon, 1990; Barbieri et al., 2004; Kopsell et al., 2005; Deschamps and Simon, 2006; Raimondi et al., 2006; Politeo et al., 2007; Chalchat and Ozcan, 2008; Hussain et al., 2008; Klimánková et al., 2008; Olfati et al., 2012; Tarchoune et al., 2013; Bantis et al., 2016).

Basil is a highly valued, annual culinary and medicinal herb that has a complex aroma profile preferred by top restaurants and professional chefs. Basil is rich in antioxidants and phenolic compounds that have been shown to possess human health benefits (Kopsell et al., 2005; Kopsell and Sams, 2013; Kim et al., 2016). The Genovese cultivar is considered valuable because of its popularity and highly desirable flavor/aroma profiles, with extensive use in culinary dishes and manufacturing productions (i.e., essential oils, soaps, etc.). In comparison to other herbs, basil contains a wide variety of compounds that are nutritionally significant to humans, including terpenes, flavonoids, carotenoids, volatile compounds, etc. (Bourgaud et al., 2001; Hussain et al., 2008). In previous surveys, basil has been reported as having one of the highest antioxidant concentrations compared to other popular herbs and spices (Kopsell et al., 2005; Lee et al., 2005; Politeo et al., 2007), increasing potential health benefits for consumers. To date, only a few studies have been conducted using a supplemental dichromatic light-emitting diode (LED) lighting source to influence basil sensory quality and VOC flavor profiles across seasons.

The advancement of LED technology provides researchers the opportunity to investigate how narrowband wavelengths fundamentally impact plant metabolism and development (Darko et al., 2014). Purposefully manipulating spectral quality in order to promote increases in secondary metabolic production has been shown to increase the nutritional value of specialty herb crops and benefit overall consumer health (Lefsrud et al., 2006; Kopsell et al., 2015; Bantis et al., 2016; Kopsell et al., 2017). Both B and R wavelengths evaluated in this study were chosen because they closely match absorption spectrums of chlorophyll and carotenoid compounds; they have direct impacts on photosynthesis and promote significant increases in overall biomass accumulation across a variety of plant species. In addition, secondary metabolic pathways may be influenced by the supplementation of specific B and R wavelengths (Colquhoun et al., 2013; Carvalho et al., 2016). These various metabolic pathways are interconnected with primary metabolism, which has the potential to impart a variety of antagonistic and synergistic effects on metabolic products that have sensory/nutritional value for humans (Kesselmeier and Staudt, 1999; Lee et al., 2005; Deschamps and Simon, 2006). A recent report indicates that basil plants grown under various narrowband wavelengths had significant concentration changes in specific volatile classes and that LED narrowband lighting may manipulate specific secondary metabolic pathways of basil under certain conditions (Carvalho et al., 2016). Another study indicated that B and R light significantly increased a variety of volatile fatty acid derivatives, volatile phenylpropanoids/benzenoids, and volatile terpenes in preharvest/postharvest tea leaves, including key flavor volatiles such as linalool, eugenol, 2-phenylethanol, etc. (Fu et al., 2015), all of which share similar pathways across species and prove relevant for the flavor and aroma of basil.

A wide range of narrowband wavelengths should be further investigated to determine potential impacts across many plant species, in addition to full-spectrum light sources. This will provide necessary information to develop effective “light recipes” that can be added for optimal growth across a variety of plant species in unfavorable growing seasons. Conflicting results have been found in relation to the suggested ratios, intensities, and daily light integral (DLI) of narrowband blue/red wavelengths for optimal plant growth and secondary metabolite production, indicating a need to further investigate the interaction between supplemental lighting (SL) and plant physiology and morphology.

The primary objective of this study was to determine the impact of progressive B/R supplemental narrowband wavelength ratios on the production of key flavor volatiles and sensory quality of basil under greenhouse conditions. Emphasis was placed on determining the optimal ratio of B/R wavelengths for VOC bioaccumulation and how changes in spectral quality/DLI impact secondary metabolic resource partitioning. Because primary and secondary metabolism are directly linked to the intensity and spectral quality of available natural light (NL), seasonal differences are expected for primary and secondary metabolism (Banthorpe et al., 1972; Kang et al., 2009; Wink, 2010; Sugimoto et al., 2012; Olle and Viršile, 2013; Carvalho et al., 2016); however, few studies have determined the relationship between specific B/R wavelengths, key flavor volatiles in basil, and impact of changing DLI/spectral quality across growing seasons in addition to narrowband SL treatments (Wink, 2010; Buchanan et al., 2015).

## Materials and Methods

### Cultural Techniques and Environmental Growing Conditions

This project was conducted at The University of Tennessee Institute of Agriculture in Knoxville, TN (35°56′44.5″N, 83°56′17.3″W). Growing dates for six separate experimental cycles occurred from August 2015 to June 2016 and have been labeled as growing seasons. “Genovese” basil seeds (Johnny’s Select Seeds, Winslow, ME, United States) were germinated in peat moss based cubes (2 × 2 × 6 cm) (Park’s Bio Dome Sponges, Hodges, SC, United States) at 28.3°C and 95% RH. The “Genovese” variety of sweet basil was specifically chosen because of its unique flavor profile, high market demand, and preference among professional chefs. After 2 weeks, seedlings were transplanted into 8 × 8 × 9-cm plastic pots using 1 part peat moss (Black Gold Canadian Sphagnum Peat Moss, Agawam, MA, United States) to 3 parts perlite (Krum Horticultural Perlite, Hodgkins, IL, United States) potting mix. Relative humidity during the growth period averaged 55%. Across all six growing seasons, day temperatures averaged 27.4°C, whereas night temperatures averaged 21.8°C. Daily and nightly temperature averages were taken across 1 full year (August 2015 to August 2016) to determine capacity of greenhouse heating/cooling systems and averaged 29.4°C/23.8°C, respectively. The DLI of the NL control averaged 9.5 mol ⋅ m^−2^ ⋅ d^–1^ across all growing cycles (ranging from 4 to 18 mol ⋅ m^−2^ ⋅ d^–1^). Specific growing parameters for each growing season may be found in Table 1.

**TABLE 1:** Important environmental parameters across growing cycles.

	September	November	January	March	April	June
**Growing season**	8/14/15–9/29/15	10/14/15–11/19/15	12/16/15–01/29/16	2/10/16–3/15/16	3/11/16–4/28/16	5/16/16–6/23/16
Average day temp (°C)	27.33	26.84	25.83	25.85	26.95	27.84
Average night temp (°C)	23.61	20.62	19.11	21.01	21.94	22.56
Average relative humidity	55%	55%	50%	55%	55%	60%
Average NL Daily Light integral (DLI) (mol ⋅ m^–2^ ⋅ d^–1^)	9.81	3.26	4.65	8.62	11.99	14.55
Average day length (h)	12.98	10.35	9.93	11.65	13.20	14.27
Average natural blue 447 nm (±10 nm) intensity at noon (μmol ⋅ m^–2^ ⋅ s^–1^)	13.8	12.2	10.3	12.1	13.3	14.5
Average natural red 627 nm (±10 nm) intensity at noon (μmol ⋅ m^–2^ ⋅ s^–1^)	14.8	13.2	10.8	13.5	14.4	15.6

*All crops grown under greenhouse conditions at The University of Tennessee Institute of Agriculture in Knoxville, TN, United States (35°56′44.5″N, 83°56′17.3″W).*

**FIGURE 1:**
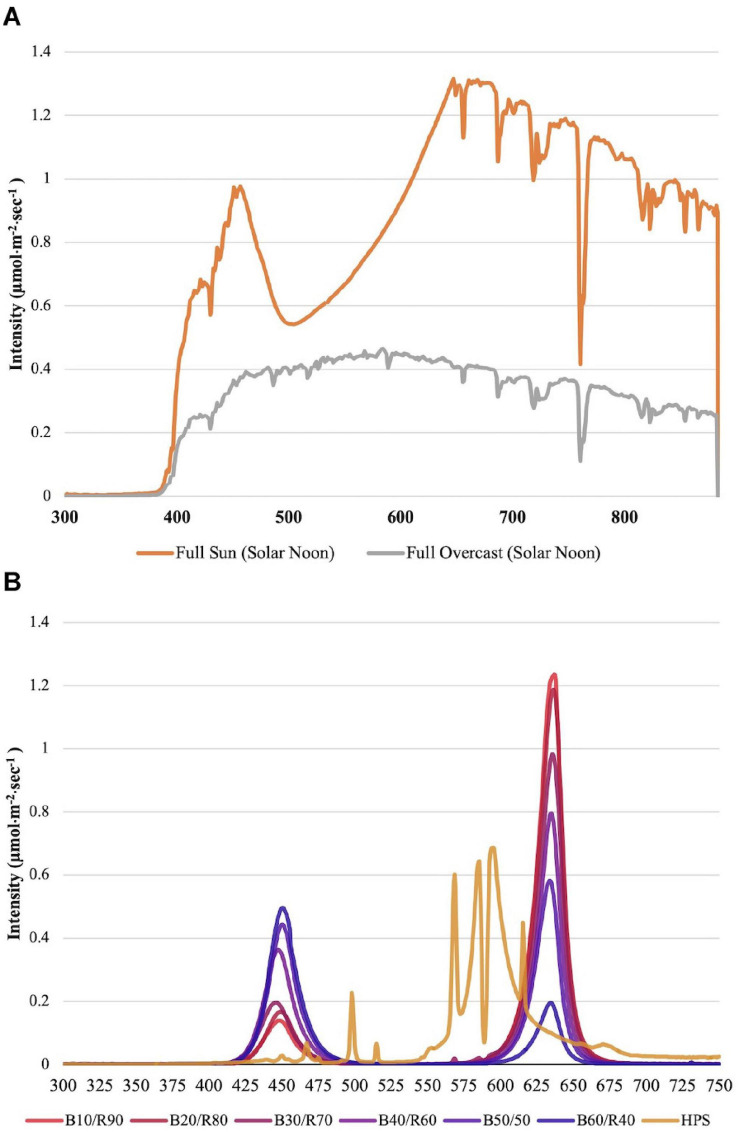
**(A)** Natural light spectra in greenhouse averaged across all six growing seasons, ranging from 300 to 875 nm. Values were taken at solar noon with three replicates for full sun and overcast for each experimental run. The daily light integral (DLI) of the NL control averaged 9.5 mol ⋅ m^−2^ ⋅ d^−1^ across all growing cycles (ranging from 4 to 18 mol ⋅ m^−2^ ⋅ d^−1^). **(B)** Emission spectra of LED and HPS treatments from 300 to 750 nm. All supplemental lighting (SL) treatments provided exactly 8.64 mol ⋅ m^−2^ ⋅ d^−1^ (100 μmol ⋅ m^–2^ ⋅ s^–1^, 24 h ⋅ d^–1^). All lighting treatments were measured with a PS-200 Apogee Spectroradiometer to confirm intensity of specific treatment wavelengths throughout all growing seasons. Readings were taken at midnight in order to exclude underlying natural solar spectra. LED treatments provided narrowband progressive blue (B)/red (R) lighting ratios (10B/90R, 20B/80R, 30B/70R, 40B/60R, 50B/50R, 60B/40R, expressed as % artificial light intensity), while the high pressure sodium (HPS) treatment provided broadband light.

Changes to key flavor volatiles were evaluated in response to specific ratios of narrowband B/R (peaked at 447 nm/627 nm, ±20 nm) LED light. Light emission spectra from natural sunlight (Figure 1A) and all SL treatments (Figure 1B) were taken across growing seasons with an Apogee PS-200 Spectroradiometer (Apogee, Logan, UT, United States). The NL control was used to establish baseline growth and development parameters under non-supplemented conditions as well as determine changes to plant physiology in response to variations in spectral quality/intensity across growing seasons. Table 1 shows average DLIs across growing season as well as the average NL intensity of B/R narrowband wavelengths that were evaluated in this experiment (447 nm/627 nm). A total of seven SL treatments were applied immediately after seedling transplant: one HPS treatment and six LED treatments with progressive B/R ratios [10B/90R, 20B/80R, 30B/70R, 40B/60R, 50B/50R, and 60B/40R (Orbital Technologies, Madison, WI, United States)]. Each SL treatment provided 8.64 mol ⋅ m^−2^ ⋅ d^–1^ across all growing seasons (100 μmol ⋅ m^–2^ ⋅ s^–1^, 24 h ⋅ d^–1^).

**FIGURE 2:**
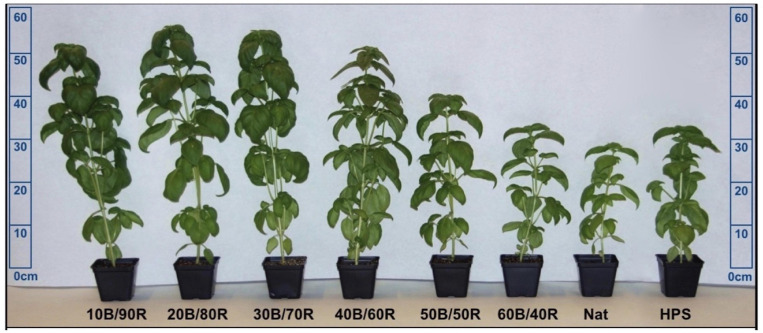
Visual representation of LED lighting impacts on morphology of hydroponically grown “Genovese” basil (*Ocimum basilicum* var. “Genovese”). Photo taken immediately before June season harvest and shows comparison of variations in height, canopy size, leaf area, and pigmentation after being exposed to supplemental blue (B)/red (R) LED ratios, natural light (NL) control (Nat), and broad-spectrum HPS supplemental lighting (SL). A non-supplemented NL control was used to account for daily light integral (DLI) and spectral quality variations across seasons. All treatments (except control) were provided 100 μmol ⋅ m^–2^ ⋅ s^–1^ of supplemental light from transplant to harvest.

Basil plants were grown in ebb and flow hydroponic systems and subirrigated for 5 min each day with a full-strength, modified Hoagland’s solution. Nutrient solution elemental concentrations were as follows (ppm): N (207.54), P (50.87), K (298.23), Ca (180.15), Mg (77.10), S (136.45), Fe (3.95), Mn (0.90), Zn (0.40), Mo (0.09), Cu (0.90), and B (0.90). The fertility regimen was kept constant across the duration of all seasons. Total growth time lasted approximately 45 days across all six experimental cycles. Like many commercial basil-growing operations, harvest occurred as the first signs of change from vegetative to reproductive growth were observed (i.e., 8–10 nodes). Figure 2 provides a visual representation of morphological and developmental changes imparted by SL treatments.

### Gas Chromatography–Mass Spectrometry Headspace Volatile Analysis

Three grams of fresh leaf tissue (two basil plants per sample rep, 1.5 g of representative material from each plant, nodes 4 and 8) were placed in 20-mL borosilicate glass vials then immediately sealed and placed onto a Network Headspace Sampler (Agilent G1888, Santa Clara, CA, United States). Six sample reps were used per treatment. Samples were heated to 80°C for 10 min and then pressurized with helium (Air Gas, analytical purity) to 95.21 kPa for 1 min. The tube was then vented for 1 min into the headspace transfer line (110°C) and injected (port at 250°C) into the gas chromatography (GC) (Agilent Technologies 6890N Network GC System). The volatiles were separated by an HP-5MS capillary column [(5%-phenyl)-methylpolysiloxane, length: 30 m, internal diameter: 0.250 mm, film thickness: 1 μm, Agilent Technologies)] using analytical purity helium carrier gas at 95.21 kPa with constant column pressure. At the start of data acquisition, temperature was held at 40°C for 5 min, ramped up from 40 to 250°C (5°C per min), and then held constant for the duration of the run. Total run time was 70 min, including post-run and cool-down phases. After sample separation and column elution, the analytes were passed through a mass selective detector (Agilent Technologies 5973 Network Mass Selective Detector) at 250°C and collected over the course of the sample run. The transfer line, ion source, and quadrupole temperatures were 250, 230, and 170°C, respectively. The full scan mass range was set to 40–550 m/z (threshold: 150).

Agilent ChemStation was used for data collection and processing. More than 200 separate compounds were identified throughout this experiment, but emphasis was placed on key flavor compounds (i.e., shown in the literature to be important for human sensory perception) that have been calibrated to our GC–mass spectrometry (MS) and HP-5MS column using analytical standards (Sigma–Aldrich, St. Louis, MO, United States) to determine leaf tissue concentrations of key VOCs on a fresh plant weight basis. The MS spectra from analytical standards and fresh samples were compared to NIST, ADMIS, and our custom basil reference library created from calibrated analytical standards to confirm peak identity and retention times. MassHunter Workstation Software Version B.06.00 (Agilent Technologies, Inc., 2012) was used to automatically integrate peaks. Relative peak areas were automatically adjusted based on analytical standards and multiple library references.

### Statistical Analyses

A randomized complete block design was used for this experiment. All data sets were analyzed by generalized linear model and mixed-model analysis of variance procedures using the statistical software SAS (version 9.4, SAS Institute, Cary, NC, United States). Design and Analysis macro (DandA.sas), created by Dr. Arnold Saxton, was utilized in addition to Tukey adjustment, regression analysis, and univariate/normalization procedures to provide additional statistical insights on the complete data set. Treatments were separated by least significant difference (LSD) at *α* = 0.05. Because of the overwhelming number of compounds analyzed, only statically significant separations were reported from this study. Key volatiles were analyzed and presented on a fresh mass (FM) basis in comparison to micromolar calibration curves created from analytical standards. All volatile concentrations units are reported in micromolarity of analyte concentration per g of fresh leaf tissue (μM . g^–1^ FM) to most accurately represent VOC emissions from the collected headspace sample above fresh plant tissues under specific reproducible analytical conditions.

## Results

**FIGURE 3:**
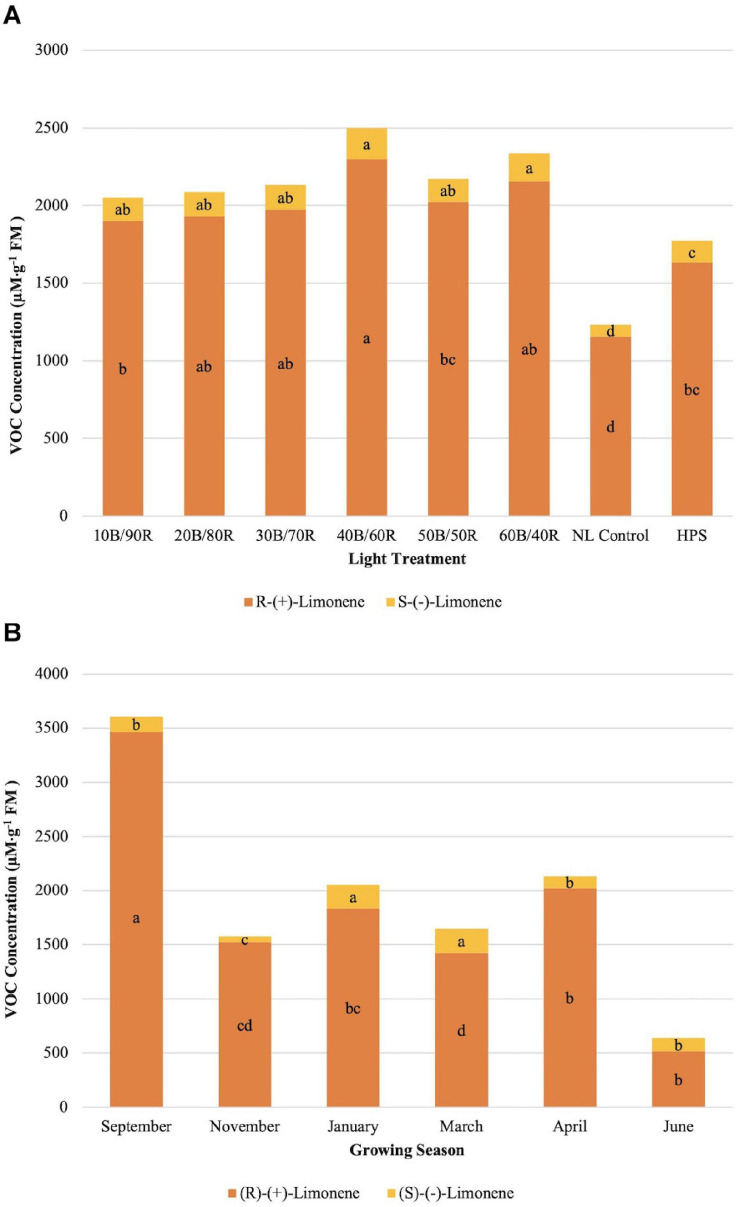
Influence of **(A)** greenhouse lighting treatments and **(B)** growing season on (R)-(+)-limonene and (S)-(−)- Limonene concentrations (μM ⋅ g^–1^ FM) of hydroponically grown “Genovese” basil (*Ocimum basilicum* var. “Genovese”). Six LED treatments with progressive blue (B)/red (R) ratios (10B/90R, 20B/80R, 30B/70R, 40B/60R, 50B/50R, 60B/40R, expressed as % artificial light intensity), and the high-pressure sodium (HPS) treatment provided supplemental light at 8.64 mol ⋅ m^−2^ ⋅ d^−1^ (100 μmol ⋅ m^–2^ ⋅ s^–1^, 24 h ⋅ d^–^1). The daily light integral (DLI) of the NL control averaged 9.5 mol ⋅ m^−2^ ⋅ d^−1^ during the growth period (ranging from 4 to 18 mol ⋅ m^−2^ ⋅ d^−1^). Supplemental lighting was then compared to the natural light (NL) control. Values were analyzed using Tukey protected LSD, and those followed by the same letter are not significantly different (α = 0.05).

(R)-(+)-limonene concentrations were significantly different across season (*P* ≤ 0.0001; *F* = 115.43), treatment (*P* ≤ 0.0001; *F* = 14.90), and season × treatment interaction (*P* = 0.0050; *F* = 1.76). (S)-(−)-limonene concentrations were significantly impacted by season (*P* ≤ 0.0001; *F* = 52.86), treatment (*P* ≤ 0.0001; *F* = 14.03), and season × treatment interaction (*P* ≤ 0.0001; *F* = 2.32). The optimal LED treatment for increasing both limonene compound concentrations was 40B/60R (Figure 3A). In general, (S)-(−)-limonene and (R)-(+)-limonene concentrations differed by a factor of 7–10 times across treatments, with (R)-(+)-limonene being the more intense flavor volatile in terms of analytical response (Figure 3A). Concentration ratios of (S)-(−)-limonene and (R)-(+)-limonene were not significantly different across all lighting treatments, which demonstrates that varying B/R light treatments do not modify the ratio of (S)-(−)-limonene and (R)-(+)-limonene produced, but rather the quantity. (S)-(−)-limonene concentrations were highest during the winter growing seasons (January–March) (Figure 3B). Average natural DLIs were much lower during this time in comparison to other growing seasons, which shows that the supplemental B/R wavelengths have greater impact on secondary metabolism when DLI is lower for this specific compound. November had the lowest concentration of overall terpenoid compounds across all seasons. (R)-(+)-limonene did not follow the same trend across growing seasons as its enantiomer (Figure 3B); ratios of R/S limonene varied across all growing seasons and did not follow any definitive patterns. September showed the highest concentrations of (R)-(+)-limonene, a six times increase over June, which was the lowest concentration observed across all growing seasons.

**FIGURE 4:**
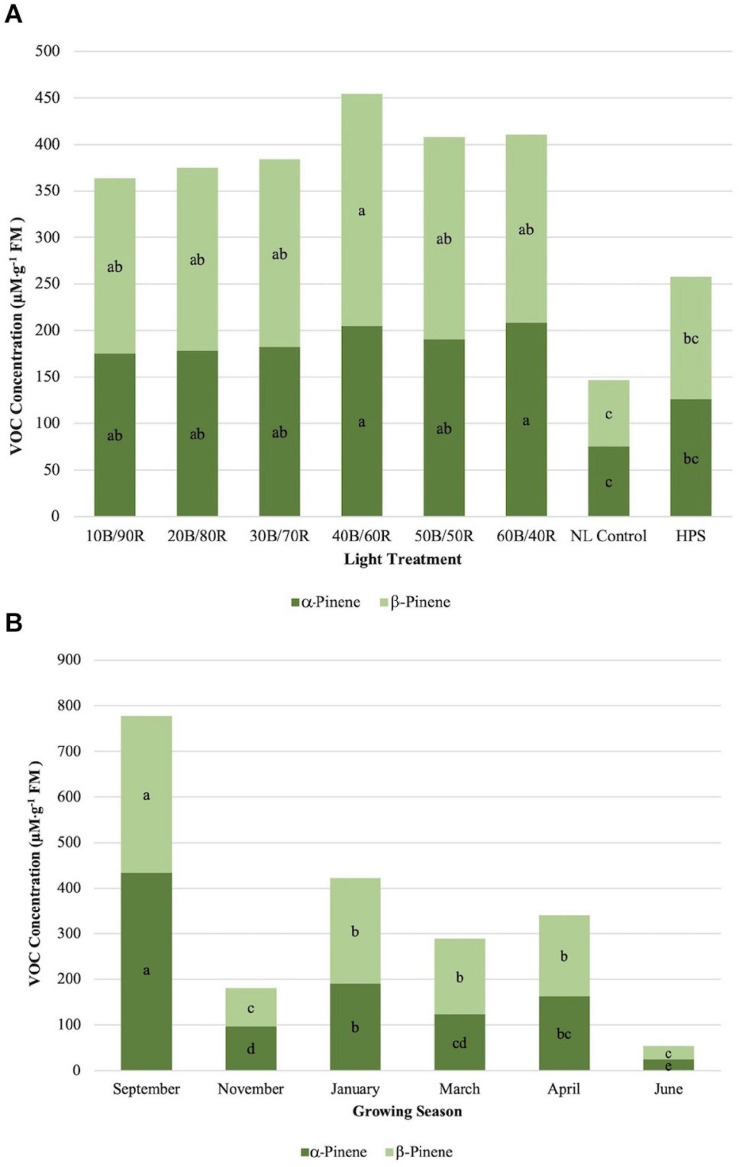
Influence of **(A)** greenhouse lighting treatments and **(B)** growing season on α-pinene and β-pinene concentrations (μM ⋅ g^–1^ FM) of hydroponically grown “Genovese” basil (*Ocimum basilicum* var. “Genovese”). Six LED treatments with progressive blue (B)/red (R) ratios (10B/90R, 20B/80R, 30B/70R, 40B/60R, 50B/50R, 60B/40R, expressed as % artificial light intensity), and the high-pressure sodium (HPS) treatment provided supplemental light at 8.64 mol ⋅ m^−2^ ⋅ d^−1^ (100 μmol ⋅ m^–2^ ⋅ s^–1^, 24 h ⋅ d^–^1). The daily light integral (DLI) of the NL control averaged 9.5 mol ⋅ m^−2^ ⋅ d^−1^ during the growth period (ranging from 4 to 18 mol ⋅ m^−2^ ⋅ d^−1^). Supplemental lighting was then compared to the natural light (NL) control. Values were analyzed using Tukey protected LSD, and those followed by the same letter are not significantly different (α = 0.05).

α-Pinene concentrations were significantly impacted by season (*P* ≤ 0.0001; *F* = 56.98), treatment (*P* ≤ 0.0001; *F* = 9.46), and season × treatment interaction (*P* ≤ 0.0010; *F* = 1.96). All LED treatments were significantly higher than the NL control. HPS treatment concentration was not significantly different than the control and did not separate from some of the LED treatments (Figure 4A). The highest concentrations were found in early fall, whereas the lowest concentrations were found in late spring, both significantly different than any other growing season (Figure 4B). β-Pinene concentrations were significantly impacted by season (*P* ≤ 0.0001; *F* = 42.03), treatment (*P* ≤ 0.0001; *F* = 10.25), and season × treatment interaction (*P* = 0.0017; *F* = 1.90). All LED treatments were significantly higher than the NL control. HPS treatment was not significantly different than the control and did not separate from some of the LED treatments. The LED treatments show a regression trend, with 40B/60R being the optimal B/R ratio for pinene bioaccumulation (Figure 4A). The highest concentrations were found in early fall, whereas the lowest concentrations were found in early winter and late spring; late spring had significantly lower concentrations, almost five times difference than the best season (September) (Figure 4B). Concentration totals of limonene and pinene compounds followed similar patterns to one another across season.

**FIGURE 5:**
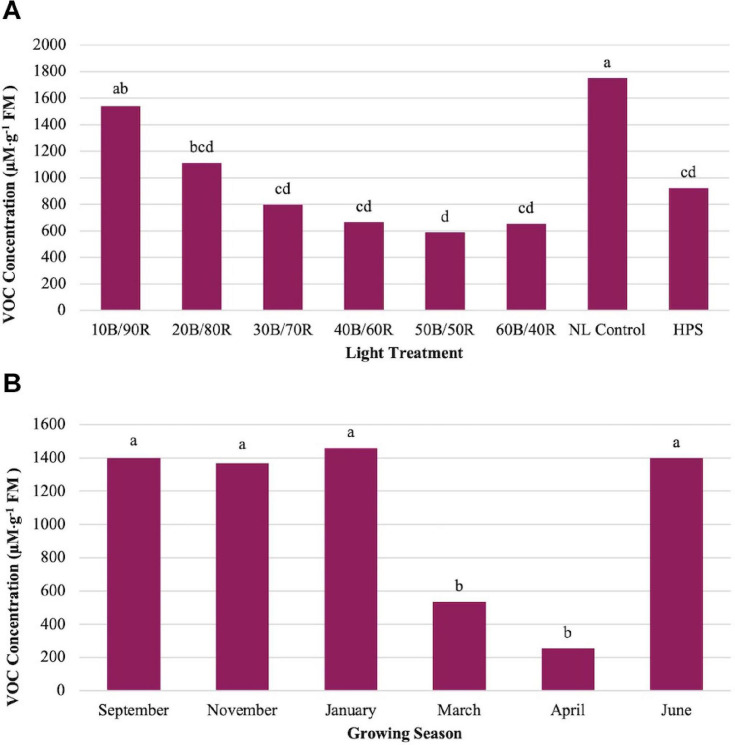
Influence of **(A)** greenhouse lighting treatments and **(B)** growing season on Methyl Eugenol concentrations (μM ⋅ g^–1^ FM) of hydroponically grown “Genovese” basil (*Ocimum basilicum* var. “Genovese”). Six LED treatments with progressive blue (B)/red (R) ratios (10B/90R, 20B/80R, 30B/70R, 40B/60R, 50B/50R, 60B/40R, expressed as % artificial light intensity), and the high-pressure sodium (HPS) treatment provided supplemental light at 8.64 mol ⋅ m^−2^ ⋅ d^−1^ (100 μmol ⋅ m^–2^ ⋅ s^–1^, 24 h ⋅ d^–^1). The daily light integral (DLI) of the NL control averaged 9.5 mol ⋅ m^−2^ ⋅ d^−1^ during the growth period (ranging from 4 to 18 mol ⋅ m^−2^ ⋅ d^−1^). Supplemental lighting was then compared to the natural light (NL) control. Values were analyzed using Tukey protected LSD, and those followed by the same letter are not significantly different (α = 0.05).

Methyl eugenol (ME) showed significant impacts across season (*P* ≤ 0.0001; *F* = 23.48), treatment (*P* ≤ 0.0001; *F* = 14.14), and season × treatment interaction (*P* ≤ 0.0001; *F* = 2.37). ME showed an inverse relationship to LED lighting treatments and NL control in comparison to other key flavor volatiles evaluated in this study (Figure 5A). Many of the compounds evaluated (i.e., isoprenoids) show a regression relationship between the LED lighting treatments peaking around 40B/60R and the NL control showing the lowest concentrations. The opposite was true for ME, as the NL control showed the highest concentrations in comparison to any other treatment, whereas the 40B/60R had the lowest concentrations. March and April had the statistically lower concentrations of ME as compared to the four other seasons (Figure 5B).

**FIGURE 6:**
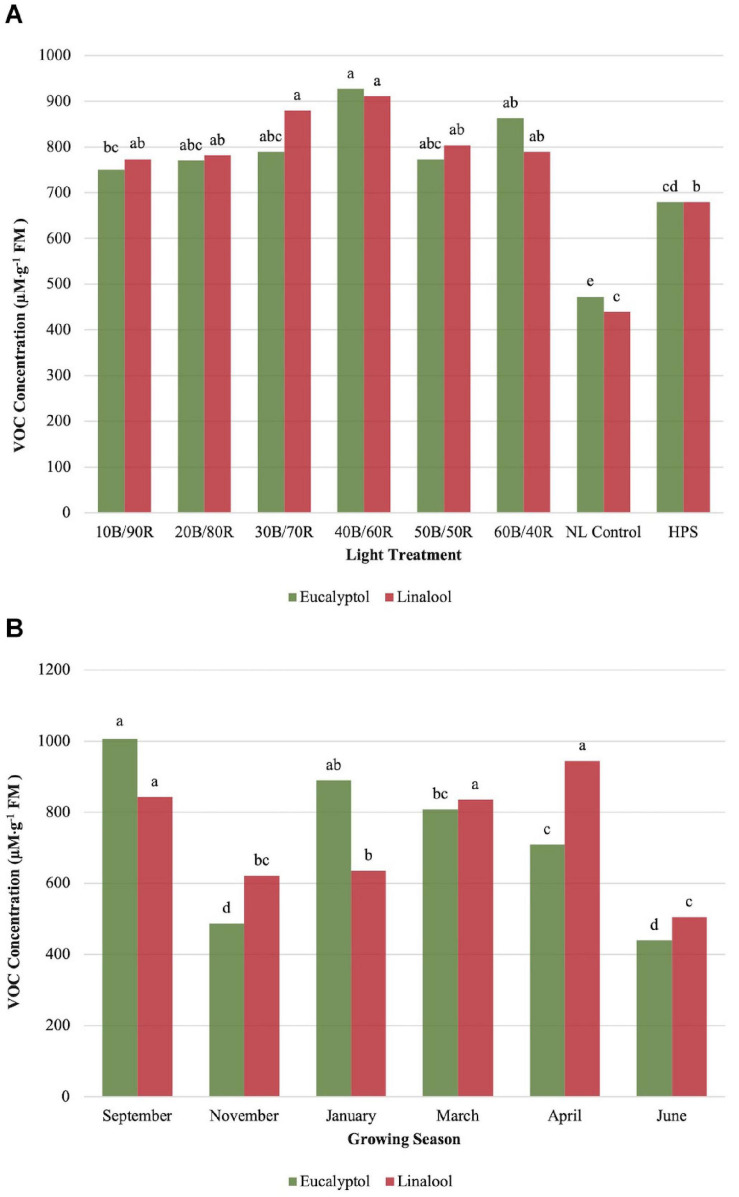
Influence of **(A)** greenhouse lighting treatments and **(B)** growing season on eucalyptol and linalool concentrations (μM ⋅ g^–1^ FM) of hydroponically grown “Genovese” basil (*Ocimum basilicum* var. “Genovese”). Six LED treatments with progressive blue (B)/red (R) ratios (10B/90R, 20B/80R, 30B/70R, 40B/60R, 50B/50R, 60B/40R, expressed as % artificial light intensity), and the high-pressure sodium (HPS) treatment provided supplemental light at 8.64 mol ⋅ m^−2^ ⋅ d^−1^ (100 μmol ⋅ m^–2^ ⋅ s^–1^, 24 h ⋅ d^–^1). The daily light integral (DLI) of the NL control averaged 9.5 mol ⋅ m^−2^ ⋅ d^−1^ during the growth period (ranging from 4 to 18 mol ⋅ m^−2^ ⋅ d^−1^). Supplemental lighting was then compared to the natural light (NL) control. Values were analyzed using Tukey protected LSD, and those followed by the same letter are not significantly different (α = 0.05).

Eucalyptol concentrations were impacted by season (*P* ≤ 0.0001; *F* = 47.47), treatment (*P* ≤ 0.0001; *F* = 16.20), and season × treatment interaction (*P* = 0.0007; *F* = 2.00). All LED treatment concentrations were higher than the NL control. HPS treatment showed some separation for optimal LED treatments, but did not separate from other LED treatments. 40B/60R produced the highest eucalyptol concentrations, with nearly 1-mM emission per gram of fresh leaf tissue. The HPS treatment separated from the NL control (Figure 6A). Early fall concentrations were higher than winter and spring seasons and generally followed the pattern observed by other monoterpenes evaluated in this experiment (Figure 6B).

Linalool concentrations were significantly impacted by season (*P* ≤ 0.0001; *F* = 28.24), treatment (*P* ≤ 0.0001; *F* = 16.52), and season × treatment interaction (*P* = 0.0398; *F* = 1.48). Concentrations peaked around 40B/60R, and this lighting treatment did not separate from other LED lighting treatments. All LED treatments were significantly higher than NL control (Figure 6A). September, March, and April had statistically higher concentrations than the other three seasons (Figure 6B).

**FIGURE 7:**
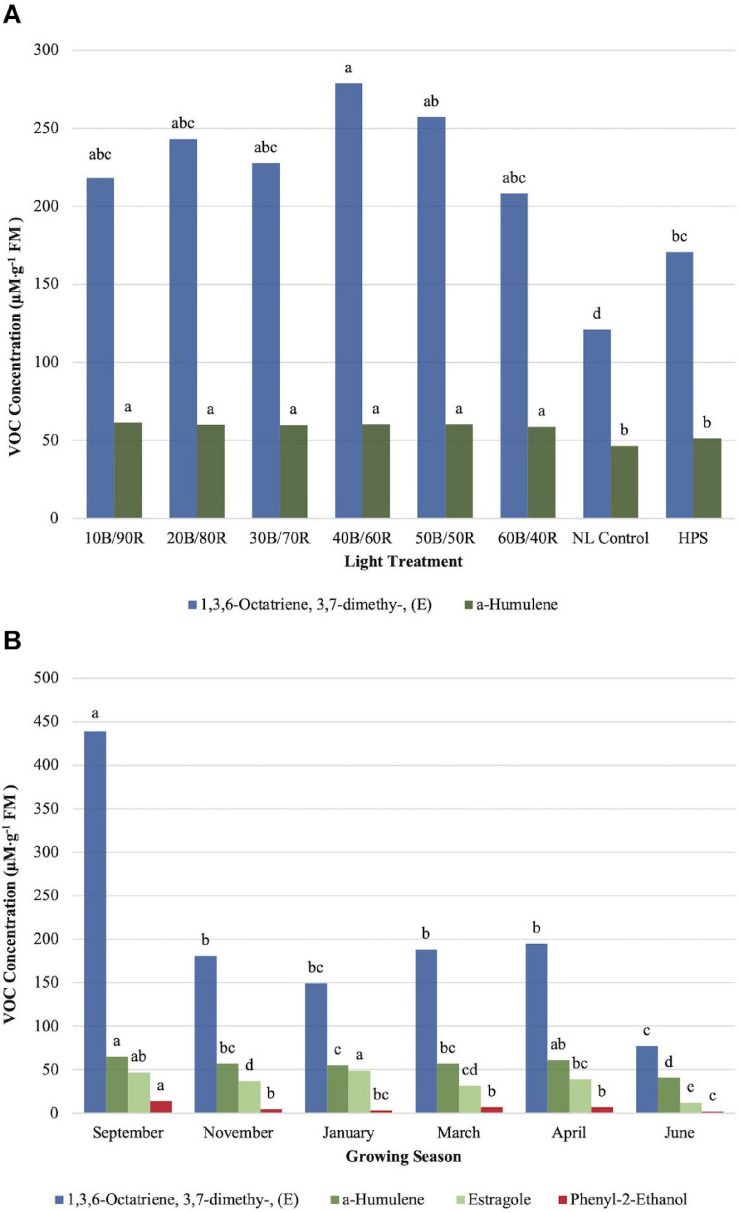
Influence of **(A)** greenhouse lighting treatments and **(B)** growing season on moderate concentration VOCs (μM ⋅ g^–1^ FM) of hydroponically grown “Genovese” basil (*Ocimum basilicum* var. “Genovese”). Six LED treatments with progressive blue (B)/red (R) ratios (10B/90R, 20B/80R, 30B/70R, 40B/60R, 50B/50R, 60B/40R, expressed as % artificial light intensity), and the high-pressure sodium (HPS) treatment provided supplemental light at 8.64 mol ⋅ m^−2^ ⋅ d^−1^ (100 μmol ⋅ m^–2^ ⋅ s^–1^, 24 h ⋅ d^–^1). The daily light integral (DLI) of the NL control averaged 9.5 mol ⋅ m^−2^ ⋅ d^−1^ during the growth period (ranging from 4 to 18 mol ⋅ m^−2^ ⋅ d^−1^). Supplemental lighting was then compared to the natural light (NL) control. Values were analyzed using Tukey protected LSD, and those followed by the same letter are not significantly different (α = 0.05).

α-Humulene concentrations were significantly impacted by season (*P* ≤ 0.0001; *F* = 34.34), treatment (*P* ≤ 0.0001; *F* = 12.29), and season × treatment interaction (*P* ≤ 0.0001; *F* = 3.20). All LED treatments were significantly higher than the NL control and HPS treatment; however, none of the LED treatments separated from one another, and the control/HPS treatment did not separate (Figure 7A). Volatile emission from LED treatments was approximately 10 μM . g^–1^ FM higher than control and HPS. Linalool and α-humulene concentrations showed significance across growing seasons, with the highest concentrations in September, March, and April (Figures 6B, 7B). The lowest concentrations for both isoprenoid compounds were found in June.

1,3,6-Octatriene,3,7- dimethy-,(E) showed significant season (*P* ≤ 0.0001; *F* = 40.80) and treatment (*P* ≤ 0.0001; *F* = 5.99) differences. The (Z) conformation did not show any significant changes across lighting treatments and was not presented in this study. The (E) conformation did show significant differences between the 40B/60R treatment and the HPS treatment/NL control (Figure 7A). Approximately 1.8 times concentration increase was observed for the (E) conformation between the best LED treatment (40B/60R) and the NL control. The concentration of the 40B/60R treatment was higher than the HPS treatment; the HPS treatment did not separate from most of the other LED treatments but did separate from the NL control. The (E) conformation of 1,3,6-octatriene showed seasonal concentration changes, but the (Z) conformation did not show any significant concentration changes across season. September had the highest concentrations, six times higher than the June growing season, which had the lowest concentration of any growing season observed (Figure 7B).

Estragole concentrations were significantly impacted by season (*P* ≤ 0.0001; *F* = 36.57) and season × treatment interaction (*P* = 0.0017; *F* = 1.89) but did not show significant impacts across treatments (*P* = 0.1127; *F* = 1.64). Midwinter and early fall had the highest levels of estragole, whereas late spring/early summer showed significantly lower levels than all the other seasons (Figure 7B).

**FIGURE 8:**
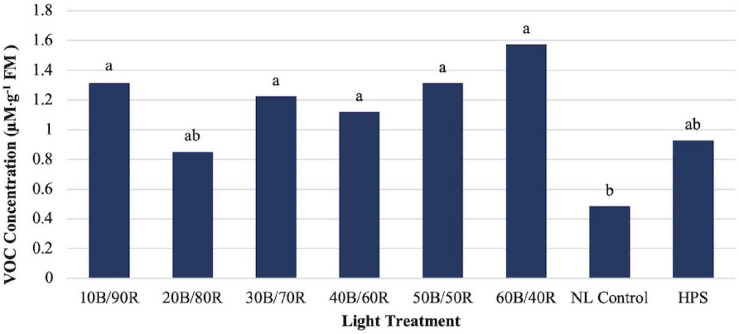
Influence of greenhouse lighting treatments on hexanal concentrations (μM ⋅ g^–1^ FM) of hydroponically grown “Genovese” basil (*Ocimum basilicum* var. “Genovese”). Six LED treatments with progressive blue (B)/red (R) ratios (10B/90R, 20B/80R, 30B/70R, 40B/60R, 50B/50R, 60B/40R, expressed as % artificial light intensity), and the high-pressure sodium (HPS) treatment provided supplemental light at 8.64 mol ⋅ m^−2^ ⋅ d^−1^ (100 μmol ⋅ m^–2^ ⋅ s^–1^, 24 h ⋅ d^–^1). The daily light integral (DLI) of the NL control averaged 9.5 mol ⋅ m^−2^ ⋅ d^−1^ during the growth period (ranging from 4 to 18 mol ⋅ m^−2^ ⋅ d^−1^). Supplemental lighting was then compared to the natural light (NL) control. Values were analyzed using Tukey protected LSD, and those followed by the same letter are not significantly different (α = 0.05).

Hexanal concentrations were significantly impacted by season (*P* ≤ 0.0001; *F* = 76.86), treatment (*P* ≤ 0.0001; *F* = 4.12), and season × treatment interaction (*P* = 0.0002; *F* = 2.14). Some LED treatments showed separation from the control, but concentrations were relatively low across all treatments as compared to many of the other key flavor volatiles (Figure 8).

**FIGURE 9:**
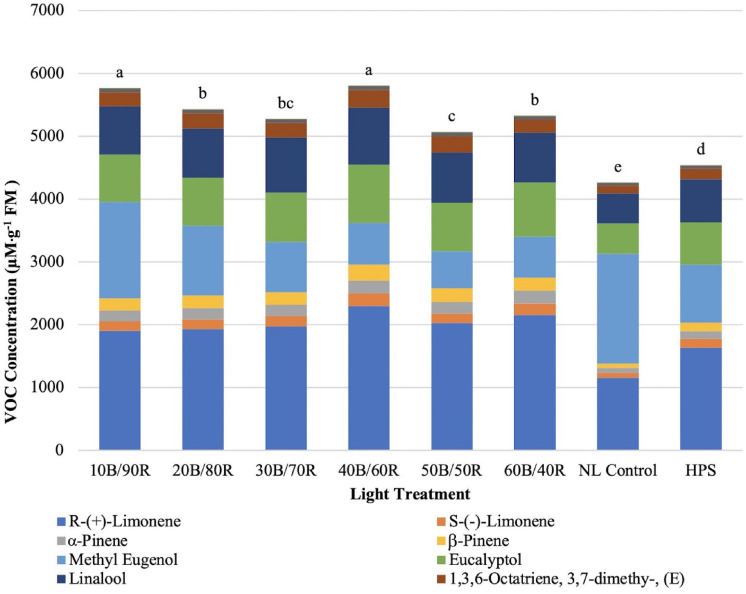
Influence of greenhouse lighting treatments on total key flavor VOC concentrations (μM ⋅ g^–1^ FM) of hydroponically grown “Genovese” basil (*Ocimum basilicum* var. “Genovese”). Six LED treatments with progressive blue (B)/red (R) ratios (10B/90R, 20B/80R, 30B/70R, 40B/60R, 50B/50R, 60B/40R, expressed as % artificial light intensity), and the high-pressure sodium (HPS) treatment provided supplemental light at 8.64 mol ⋅ m^−2^ ⋅ d^−1^ (100 μmol ⋅ m^–2^ ⋅ s^–1^, 24 h ⋅ d^–^1). The daily light integral (DLI) of the NL control averaged 9.5 mol ⋅ m^−2^ ⋅ d^−1^ during the growth period (ranging from 4 to 18 mol ⋅ m^−2^ ⋅ d^−1^). Supplemental lighting was then compared to the natural light (NL) control. Values were analyzed using Tukey protected LSD, and those followed by the same letter are not significantly different (α = 0.05).

Total concentrations of key flavor volatiles analyzed in this experiment were significantly impacted by lighting treatment (*P* ≤ 0.0001; *F* = 81.21). Total concentrations of VOCs ranged from approximately 4,200–5,800 μM . g^–1^ FM (Figure 9). The highest total concentration of VOCs was under the 40B/60R and 10B/90R treatments. The HPS treatment was significantly lower than all LED treatments. The lowest concentration was under the NL control.

## Discussion

As expected, there were significant differences to VOC profiles across lighting treatments as well as growing season. Even though greenhouses protect from winter weather and poor climate conditions, outside environmental conditions have significant influence on crop production quality and yields. Some factors that may impact volatile concentrations across growing seasons include reduced NL intensity, inferior spectral quality, increased cloud cover, day/night temperature reductions and fluctuations (including difference in day/night temperature, DIF), and changes in relative humidity. SL significantly increased collective terpenoid concentrations during winter months. SL treatments caused significant changes to many VOC concentrations even during seasons with much higher DLI (close to full saturation for basil) and more favorable NL spectral quality. Key flavor volatiles in basil within specific classes (terpenoids vs. phenols) of secondary metabolites had opposing changes in concentration across growing seasons and followed some general trends that coincide with many studies that investigate the impact of abiotic factors (i.e., DLI and spectral quality) on crop production and physiology (McCree, 1973; Smith, 1982; Lange et al., 2000; Briggs and Christie, 2002; Loughrin and Kasperbauer, 2003; Leonard et al., 2010; Gouvea et al., 2012; Olle and Viršile, 2013; Ouzounis et al., 2015b; Kopsell et al., 2017).

Many of the compounds explored in this study include abundant isoprenoids and phenylpropanoids found in edible tissues from basil that profoundly impact human sensory experience (i.e., flavor and aroma). (S)-(−)-limonene and (R)-(+)-limonene are two important flavor compounds that have significant influence on the sensory perception of basil. (S)-(−)-limonene is said to have an orange like aroma, whereas (R)-(+)-limonene has a stronger lemon/citrus aroma (Larsen et al., 2016). Both monoterpenes possess similar bioactivities (i.e., detection threshold and intensity of sensory response). The combination of these two limonene compounds, in addition to other volatile compounds, contributes to overall citrus flavor. Changes in the relative concentrations of these flavor volatiles determine the “type” of citrus flavor that is perceived by humans as well as the intensity. Both (S)-(−)-limonene and (R)-(+)-limonene concentrations were impacted by lighting treatment and season. This suggests that spectral quality and DLI have a direct impact on secondary pathways upstream from the R/S conformation point and produce higher volumes of both compounds (i.e., increased production of isoprene subunits required for monoterpene production).

November had the highest concentrations of total limonene compounds across all seasons, which may be explained by higher NL intensity during the first stage of development and lower NL intensity as the growing season progressed into late fall. Day/night temperature averages were maintained with a few degrees throughout the growing season. While greenhouses reduce temperature variation across growing seasons, it is nearly impossible for any type of environmental control system to completely eliminate variations in humidity and temperature across all growing seasons. Minute temperature variations are expected across growing seasons, but do not provide basis for the stark contrast between generalized LED treatment concentrations and HPS treatment concentrations. It is also possible that secondary metabolic carryover effects were experienced from the seedling stage into later growth periods. Multiple studies have found that specific wavelength, light intensities, and temperature variations at the seedling stage can impact secondary metabolism and resource partitioning for specific classes of terpenoids at later stages of development (Massa et al., 2008; Randall and Lopez, 2014; Bantis et al., 2016, 2020; Carvalho et al., 2016; Jishi et al., 2016; Matsuda et al., 2016).

Total concentrations of both compounds did not follow any physiological-based pattern across growing seasons. This is somewhat unexpected as both limonene compounds are so closely related in terms of chemical structure, biosynthesis, bioactivity in humans, and importance in flavor for basil/other herbaceous and citrus crops. This phenomenon of seasonal variation is mostly a result of the interaction between DLI and spectral quality (i.e., the ratio of supplemental/NL at different times of the year), which in turn controls a specific mechanism or pathway up-/down-regulation at the point in the metabolic pathway that determines the R/S conformation. Passive volatilization or other physical chemistry-based phenomena alone cannot explain the differences in limonene compound concentrations across treatments and seasons, as emission would theoretically be similar for both; unless a specific mechanism or active process is responsible for biosynthesizing and/or emitting one volatile over the other as a result of environmental changes. Leaf temperatures were recorded and maintained with 2°C across all treatments and growing seasons, which further reduces the likelihood that passive volatilization is responsible.

α-Pinene and β-pinene are two other monoterpenes that have significant influence on the flavor and aroma of basil, both of which are impacted by the total concentration of all pinene compounds present as well as the concentration ratio of different pinene isomers in relation to one another. Pinene compounds are primarily found in pine resin, and they are one of the most abundant terpenoids in nature (Noma et al., 2010). These compounds have high biological activities in mammals (both beneficial medical properties, as well as moderate toxicity levels) and strongly repel insects. α-Pinene and β-pinene concentrations were impacted by treatment and season. Concentration totals of limonene and pinene compounds followed similar patterns to one another across season, both of which belong to the same chemical class (monoterpenes) and are made in the same pathway deriving from isoprene subunits. Independent of lighting treatment, slightly higher levels of β-pinene were observed in comparison to α-pinene (i.e., ratio of α/β). These ratios remained consistent across lighting treatments but were significantly different across season, further suggesting that changes in both DLI and spectral quality influence secondary metabolism, and these variables have interacting effects on VOC production.

Methyl eugenol (ME) is a phenolic compound that possesses a strong, spicy, herbaceous aroma, which greatly contributes to the flavor and aroma of basil. This compound also has valuable medicinal properties and many human health benefits (Lee et al., 2005). It can be described as having a clove-like flavor and is used extensively in the pharmaceutical and cosmetic industries. This compound has been labeled as an antioxidant, antimutagenic, antigenotoxic, and anti-inflammatory and even has the potential to reduce the recurrence of certain cancers (Carvalho et al., 2016). While ME shows a clear pattern across treatments, it does not show a pattern with increasing DLI across seasons, suggesting that spectral quality has greater influence on ME concentrations when compared to DLI (Figures 5A,B). March and April growing seasons showed the lowest ME concentrations. All other growing seasons (September, November, January, June) showed elevated concentrations and did not statistically separate (Figure 5B). This was unexpected as there is less ambient NL and inferior spectral quality at the start of the November and January growing seasons as compared to the March and April season. Further, June had the highest DLI, but ME concentrations were not significantly different from the other optimal seasons. ME may be released through passive volatilization in higher quantities due to changes in light intensity and spectral quality because of its high boiling point and vapor pressure. ME is derived from a separate secondary metabolic pathway (may be derived from a variety of pathways, but dominant biosynthesis occurs from the precursor amino acid L-tyrosine and is converted into a variety of eugenol conformations through multistep biosynthesis) as compared to the other terpene-based compounds created from the isoprenoid pathway. This suggests that spectral quality has a direct impact on the resource partitioning in this secondary pathway, specifically tyrosine and other enzymes that are used to synthesize eugenol and other phenolic compounds. These results are consistent with similar studies that investigated the impact of LED lighting on flavor volatiles and resource partitioning of other secondary metabolites (Loughrin and Kasperbauer, 2003; Colquhoun et al., 2013; Samuolienė et al., 2013; Carvalho et al., 2016). Overall concentrations of terpene-based compounds showed concentration increases with the addition of B wavelengths (peaking around 40B/60R and diminishing greatly after that), while ME generally showed concentration decreases with the addition of supplementary B light, which is consistent with results from a similar study (Carvalho et al., 2016). The unexpected changes across seasons may also be a result of varying broad-spectrum light quality and wavelengths not supplemented in the B/R regiment (i.e., variations of other B/R, yellows, greens, oranges, etc.) during certain times of the year. While basil does not exhibit photoperiodic responses toward reproductive growth, many plants have a variety of metabolic processes that are regulated by the interaction of light intensity, duration, and spectral quality, all of which may have an impact on sensory perception (i.e., flavor and aroma) (Banthorpe et al., 1972; Buchanan et al., 2015; Ouzounis et al., 2015a). It is also possible that the B/R treatments had a much more pronounced impact on monoterpene synthesis during winter/early spring months from reduced day-length and light intensity/spectral quality, which diverted resources away from the pathway that synthesizes eugenol compounds. Eugenol, isoeugenol, ME, and several other isomeric compounds should be further investigated to determine the impact of wavelength and intensity on the ratios of these specific compounds and/or overall concentrations of this class of metabolic products in comparison to other classes, as they all have impacts on flavor and aroma in basil. The benefits (i.e., increase of other terpenoids that are important to flavor) that result from the addition of supplementary wavelengths may outweigh the net loss of ME and other phenolic compounds that are also important to flavor. It is important to note that ME can be somewhat toxic and unpalatable at levels found even in naturally occurring sources; reducing the amount of ME or other somewhat toxic/unpalatable compounds may give growers the opportunity to create designer flavors while optimizing the health benefits of their crop (Reverchon, 1997; Fahlbusch et al., 2003). The biological activities, sensory impacts, and overall health benefits of each key flavor/aroma compound should be further investigated to determine which compounds are most important for basil quality and human sensory perception; in turn, this information may be used to determine the optimal spectrum and intensity of SL treatments to improve flavor and aroma profiles of basil and other high-value greenhouse crops.

Eucalyptol, or 1,8-cineole, is another monoterpene that is closely related in both chemical/physical properties and structure to pinene, limonene, and other secondary metabolites produced from isoprene subunits (Buchanan et al., 2015). Eucalyptol is the primary VOC that influences flavor in a variety of basil cultivars. In general, this compound followed the same pattern as other monoterpenes evaluated in this experiment. Eucalyptol is the most abundant flavor volatile found in basil leaf tissues, accounting for greater than 50% of GC-MS response area in a variety of studies (Loughrin and Kasperbauer, 2003; Lee et al., 2005; Klimánková et al., 2008; Claudia et al., 2013; Tarchoune et al., 2013; Bantis et al., 2016; Carvalho et al., 2016). It has a spicy, energizing, camphoraceous aroma with a cooling mint-like taste. Biological activity related to this compound is extremely high, in addition to moderate toxicity levels at relatively low concentrations. For these reasons, this compound is used in extremely low concentrations when manufacturing food products and cosmetics.

The aroma of linalool can be described as sweet and floral, similar to fruity-pebbles cereal. It has been shown to possess antioxidant and anti-inflammatory properties in addition to numerous other health benefits (Randall and Lopez, 2014; Carvalho et al., 2016). Linalool is a critical component in the overall flavor of basil and many other herbaceous crops. Linalool concentrations were significant impacted by treatment and growing season. Patterns with linalool concentration changes were consistent with other terpenoids in this, as well as other studies involving basil, mint, and other high-value crops (Lee et al., 2005; Galeotti et al., 2008; Colquhoun et al., 2013; Carvalho et al., 2016). α-Humulene is one of the primary chemical constituents that results in the flavor and aroma of flowering cones of the hops plant and many other herbaceous crops. This compound and its reaction products are essential for brewing processes and are responsible for the hoppy flavor and aroma in beer and other fermented products. This sesquiterpene can be found in many other plants in the Lamiaceae family and has relative impacts on flavor and aroma in many basil cultivars/varieties (Lee et al., 2005). Linalool and α-humulene concentrations showed significance across growing seasons, with the highest concentrations in September, March, and April. The lowest concentrations for both isoprenoid compounds were found in June. As they are both created with fundamental isoprene units and found in the same pathway. Concentration levels between the two were found approximately 10:1 linalool:α-humulene and remained constant across all seasons, with less than 5% variance across all seasonal concentration ratios. This is expected, as linalool is a monoterpene and α-humulene is a sesquiterpene, both of which are synthesized primarily in the same metabolic pathway. It was also unexpected that eucalyptol and linalool did not show similar patterns across seasons, suggesting an interaction between varying DLIs and spectral qualities impacting monoterpene synthesis.

1,3,6-Octatriene,3,7- dimethy-,(Z) and 1,3,6-octatriene,3,7- dimethy-,(E) were measured across lighting treatments, both of which exhibit an herbaceous and terpene-based aroma. Limited information was available regarding sensory perception of these compounds, concentrations within basil, the impact of LED lighting treatments on these compounds, or any combination of these areas of study. Only the (E) conformation showed significant differences across treatment and season. Phenyl-2-ethanol has a pleasant floral odor and is used extensively in the food and beverage industries. Early fall concentrations were higher than any other season, whereas late spring/early summer were the lowest. Hexanal is considered an aliphatic aldehyde and is used extensively in the food industry to produce fruity flavors. It has a fruity herbaceous odor that also resembles fresh cut grass. Hexanal concentrations were significantly impacted by treatment and season. Some LED treatments showed separation from the control, but concentrations were relatively low across all treatments. Despite having relatively low concentrations as compared to other flavor volatiles in basil, they have a great importance in flavor perception because of their high biological activities in humans.

These results are consistent with our ongoing conclusion that the combination of both spectral quality and DLI influences secondary metabolism at various points in isoprenoid and phenylpropanoid pathways. Various studies that have investigated narrowband wavelengths in relation to pinene isomers, monoterpene hydrocarbon compounds, and broad secondary metabolic resource partitioning (i.e., phenylpropanoids, sesquiterpenoids, other isoprenoids, etc.) are congruent with the findings of this study (Loughrin and Kasperbauer, 2001; Loughrin and Kasperbauer, 2003; Deschamps and Simon, 2006; Klimánková et al., 2008; Colina-Coca et al., 2013; Colquhoun et al., 2013; Olle and Viršile, 2013; Samuolienė et al., 2013; Tarchoune et al., 2013; Darko et al., 2014; Ouzounis et al., 2014; Selli et al., 2014; Du et al., 2015; Fu et al., 2015; Carvalho et al., 2016).

## Conclusion

No matter the intended use, sweet basil is highly appreciated for its aroma and flavor. High yields and overall quality are critical foundations of successful commercial growing operations. A variety of quality parameters have been established for the flavor and aroma of many high-value herbaceous crops. The delicate flavor and aroma of basil are a result of the specific and complex ratios of chemical compounds produced through primary and secondary metabolic processes directly related to environmental conditions and genetic makeup (Lachowicz et al., 1996; Samuolienė et al., 2009; Ouzounis et al., 2014). The impacts of environmental stressors, growing season, and cultivar chemotype are reflected in VOC profiles, which is a direct result of changes to secondary metabolism. The results of this experiment demonstrate that manipulating spectral quality with narrowband supplements can be used to alter secondary metabolic resource allocation (i.e., flavor profile), regardless of season.

Overall, eucalyptol, (R)-(+)-limonene, linalool, and ME showed the highest calibrated concentration abundance in basil for this experiment, approximately 1–3 mM ⋅ g^–1^ FM headspace emission across a variety of seasons and treatments. All other flavor volatiles were found in lower concentrations, within the range of 1.0–0.01 mM ⋅ g^–1^ FM headspace emission. (R)-(+)-limonene, α-pinene, β-pinene, eucalyptol, and phenyl-2-ethanol showed similar concentration patterns across growing seasons (Figures 3B, 4B, 6B, 7B). September showed the highest concentrations of these compounds, whereas November and June showed the lowest concentrations. While the specific concentrations of these individual compounds found in plant tissue varied greatly, they all followed similar concentration ratios as growing seasons progressed (in contrast to phenylpropanoids). The ratios of two important flavor compounds, α-pinene and β-pinene, were similar across lighting treatments but varied across growing seasons, which demonstrates the complex interactions between spectral quality/DLI and the impact they have on secondary metabolism. The use of B/R supplements in addition to natural spectral quality and DLI has the ability to influence flavor volatile profiles and alter secondary metabolic resource partitioning in hydroponically grown sweet basil.

## Data Availability Statement

The raw data supporting the conclusions of this article will be made available by the authors, without undue reservation.

## Author Contributions

HH developed the hypothesis, experimental design, data collection, lab analysis, statistical analysis, and prepared and edited the original manuscript. DK provided input on hypothesis and experimental design, review and editing of the manuscript. CS developed the hypothesis, experimental design, data evaluation, statistical analysis, and review and editing of the manuscript. All the authors contributed to the article and approved the submitted version.

## Conflict of Interest

The authors declare that the research was conducted in the absence of any commercial or financial relationships that could be construed as a potential conflict of interest.
